# Enhanced Knittability of Paper Yarn from the Swedish Forest by Using Textile Finishing Materials

**DOI:** 10.3390/polym13213628

**Published:** 2021-10-21

**Authors:** Felicia Syrén, Gabriella Andersson Drugge, Joel Peterson, Nawar Kadi

**Affiliations:** Department of Textile Technology, Faculty of Textiles, Engineering and Business, University of Borås, 501 90 Borås, Sweden; gabriella.andersson.drugge@outlook.com (G.A.D.); joel.peterson@hb.se (J.P.); nawar.kadi@hb.se (N.K.)

**Keywords:** paper yarn, natural fibers, sustainability, knitting

## Abstract

Friction between Swedish paper yarn and needles is a limiting factor that—together with the low yarn flexibility—is hindering the knitting and use of paper yarn as a sustainable textile material. To enhance the knittability, paper yarn was coated with textile finishing materials. The effect of six different textile finishing materials used for textiles processing (three different silicone-based, wax, glycerol, and soap) was evaluated. The treatment evaluation was done by determination of the friction coefficient, tensile testing, and knitting. The friction coefficient was determined by an adaption from the ASTM D3108-07 Standard Test Method for Coefficient of Friction, Yarn to Solid Material. The adaption meant using a specially designed rig, making it possible to simulate the yarn/needle friction during the knitting process and use a tensile testing machine to determine the friction coefficient. Through using the same angle for yarn movement during the knitting process in this adaptation, the effect of the flexibility of paper on the friction coefficient is integrated. Tensile testing was performed using a Tensolab 2512A/2512C electromechanical tensile tester, and knitting tests were performed using a Stoll CMS 822 HP knit and wear flat knitting machine with the E5.2 gauge. The results show that knittability is better for the yarns with lower coefficients of friction and can also be enhanced by spraying with regular water. The tensile properties of the yarn is degraded by the treatments. The wax- and soap-treated yarns were most challenging to knit. The silicone-based and glycerol-treated yarns showed enhanced knittability, where the glycerol treatment results in more protruding fibers compared to the other treatments. All treatments reduced the roughness in the feel of the knit. The results indicate that the Swedish paper yarn can be a future sustainable complement to polyester and cotton.

## 1. Introduction

Sustainability in textiles involves a long chain of factors to consider: material production and processing, distribution of products, consumption, and disposal [1,2,3,4,5,6,7,8,9]. The textile sector is enormous and is a significant part of several countries’ national economies [3]. Consumer demands on environmental friendliness and governmental laws drive the companies to make an effort to increase their sustainability while also fulfilling other requirements such as comfort, performance, and aesthetics [3,5,6]. For textile materials’ sustainability, renewability is desired because the majority of today’s textiles are petroleum based [2,5,10]. Therefore, a lot of research is directed toward different natural fibers where the significant problems are pesticides and chemicals [2,5,9]. The processing chemicals are used for various purposes in several process steps and end up in waste water [3].

A renewable and fairly new material for textiles is paper yarn from Swedish forests. In Japan and Korea, there is a long tradition of using paper for yarn and textile products. The Japanese paper yarn is based on Manilla hemp and the Korean (Hanji yarn) is based on mulberry fibers [11,12]. Paper yarn has both advantages and disadvantages. Since it shows some good mechanical properties and is a more sustainable alternative to cotton, the demand for paper yarn has been increasing [13]. For fashion and home textiles applications, the low shrinkage and absence of pilling of paper yarn products are desirable [14]. However, there are still properties that need to be enhanced.

Paper yarn and paper yarn-based textiles usually have several of the following drawbacks: low porosity, low flexibility, low strength, low elongation, low handleability, poor knittability, high stiffness, high grip, and high rigidity [11,14,15]. The unstable twist hinders the mass production of Hanji yarn [11]. A few studies have been made attempting to improve some of the properties. Multi-walled carbon nanotubes (MWCNT) have shown improvement in abrasion resistance, bending length, water adsorption, and crease recovery [16]. When paper denim was developed in Japan in 2015 using cotton in warp and paper yarn in weft, the low stretchability of paper yarn resulted in a denim fabric of high rigidity and problems with wrinkles. These problems were improved by Park and Lee (2018) as they used a core-spun yarn together with the paper yarn in weft [17]. Several Japanese patents show that the flexibility of paper yarn can be more or less improved by wax, oil, and steaming [11]. To enhance the paper yarn properties, Murate et al. (2008) soaked the paper yarn in a NH_3_ aqueous solution and then steamed it at 170 °C [13]. This treatment affected the stretching properties and was described as shape memorization. Vasell and Ronkainen (2017) investigated the effect of a grinding process to increase the softness of the paper yarn, where the results show a limited improvement [18]. Park and Lee (2013) showed that using water in the spinning process of Hanji (traditional Korean paper) yarn results in a more even surface. The treatment also increased the breaking strain and stress and decreased the initial modulus [11].

Paper yarn differs from other regular yarns (e.g., yarn made of cotton or polyester), because in paper yarn, the pulp fibers are converted into a paper structure, which is followed by the integration of paper strips into a yarn structure. The paper strips can either be folded or twisted into yarn. The first paper spinning machines used the twisting method, and this process is still under development. The paper is moistened by water upon the twisting, so the paper must have sufficient wet strength to endure the spinning process [19]. In general, paper offers low porosity and high stiffness [11]. On the other hand, the regular yarn structure (such as continuous multifilament or spun yarn) has the configuration of fibers dictating the properties of yarns. Previous studies on yarns with staple fibers illustrate that the cohesion between the fibers is responsible for the yarn’s structural integrity and attaining the required mechanical properties [20,21].

Paper yarn is a natural cellulose-based material where the origin of the cellulose will affect the material properties. The Swedish paper industry is dominated by softwood—spruce with a tracheid length of about 3 mm—which is commonly produced through mechanical pulping [22]. The mechanical pulping leads to increased bending stiffness as the fibers are stiffer compared to those resulting from chemical pulping [23]. In addition, the paper strength is higher as the mechanical pulping increases the amount of fines (small particles), which increases the bonding area [23]. In contrast to this, Abaca and hemp (which has been used in the production of Japanese and Korean paper yarn) are examples of raw materials for special quality paper that cannot be made from wood pulp. In addition, the processing method is different, as these would be chemically pulped with soda pulping [22]. The chemical pulping in general leads to more flexible fibers [23]. In addition to the property differences due to material origin, the mechanical properties of natural materials is determined by their microstructure and impurities, and since the microscopic structure of naturally growing materials cannot be controlled, there is a larger variability in their properties compared to synthetic materials [24,25].

As previous research shows, the paper yarn properties can be affected by the paper-making process and the yarn-spinning process. It is also possible to treat the yarn before weaving or knitting. While the weaving of paper yarn has shown promising results, knitted paper yarn fabrics are not commonly studied, and the knittability of paper yarn remains low. For the knitting of paper yarn, the high friction between the yarn and needles need to be decreased.

Friction is essential for product quality and processability, and numerous finishing materials are used in the textile industry to modify it [26]. Friction is influenced by many material and environmental factors and can also depend on the combination of material and processing parameters [27,28,29]. Surface characteristics are of high importance, as friction is the force created from shearing between contacting surfaces. An increase in yarn unevenness increases the friction. An increased surface roughness can initially give a decreased friction until a point where larger surface roughness results in increasing friction [27]. There are three types of friction of yarns and sewing threads; fiber–fiber, yarn–yarn, and yarn–metal. The yarn–metal friction is governed by yarn type and machine settings [28].

Softeners have shown effects in fiber–fiber and yarn–yarn friction [27,30]. Softeners are common in fabric finishing processes for enhanced handleability and other properties. Softeners can be classified according to their ionic character: cationic, anionic, non-ionic, amphoteric, and silicone, where cationic is a common ingredient in most softeners [31]. Cationic silicone softener has reduced friction and increased the knittability of linen yarn [30]. A softener has a different effect depending on the material [31].

Lubricants reduce yarn friction [26]. They are essential in yarn and fabric processing, reducing wear, abrasion, and static electrification while ensuring strength and protection from processing-related heat. The molecular size and charge of the lubricant determine if it stays on the surface or also reaches the bulk [27]. Lubricants can usually not decrease the coefficient of friction of yarn below 0.2 [26]. They can even reduce strength and tenacity, as seen when silicone oil lubricates the fiber–fiber interface [26,30]. Up to a 13% decrease in strength has been reported in a study where water-soluble and mineral oil-based lubricants showed less reduction [32]. A common lubricant used for the reduction of yarn/metal friction is wax [33]. Paraffin wax has shown good lubricant results and can affect the uptake [26]. There are two common application methods: wet and dry. Wet means using a bath and can give 4–6% uptake for sewing threads. Dry can be a kiss roll at the rewinding of thread/yarn and give 1–2% less uptake [26,29]. Even 2% lubricant can significantly decrease the friction of threads [29]. The dry application method has shown better friction reduction compared to wet [27]. However, the required amount of lubricant depends on the material and end use [29]. An excess of lubricant at the surface can increase yarn–metal friction [26]. 

Glycerol has been used as a lubricant and softener for various fibers, yarns, and fabrics [34,35,36]. It is termed as a green solvent, similar to water, which is cheap, non-toxic, readily available, and recyclable [34,35,36,37,38,39]. Glycerol concentration in the coating of paper also showed a positive effect on tensile strength and elongation [40].

Soap has been used as a finishing material for the woven fabric to improve its frictional properties [41].

There are many lubricants and softeners that have shown good results in improving the frictional properties of various textile materials. However, as the Swedish paper yarn in this research study is a new yarn currently under development, the effect of the known textile finishing materials has not been investigated on this kind of paper yarn before. The Swedish paper yarn used for this research study will have an effect on the frictional properties during knitting, the finishing materials’ effect on the friction, and in the optimum amount of each specific finishing material. Thus, the finishing materials’ effect needs to be investigated in combination with this new material. In addition to this, the current available research on paper yarn mainly focuses on the weaving process, where this study addresses the knitting process. It should also be noted that the paper-making process as well as raw material will have an effect on the paper’s properties and inherently the paper yarn’s properties. This is why results from studies with other sorts of paper yarns could not be directly transferred to this new material—the mechanical pulping of spruce as paper for the yarn. Therefore, in this study, roll coating of six different textile finishing materials will be performed on Swedish paper yarn: silicones, waxes, glycerol, and soap. Their influence on the coefficient of friction and knittability will be evaluated along with tensile testing. If this paper yarn can be made available for knitted applications, it could help fill the need for more and sustainable textile fibers of the future. It could replace a share of the petrol-based textile materials and be a new product for the paper industry as newspapers are more and more read in digital form.

## 2. Materials and Methods

### 2.1. Yarn and Finishing Materials

**Figure 1:**
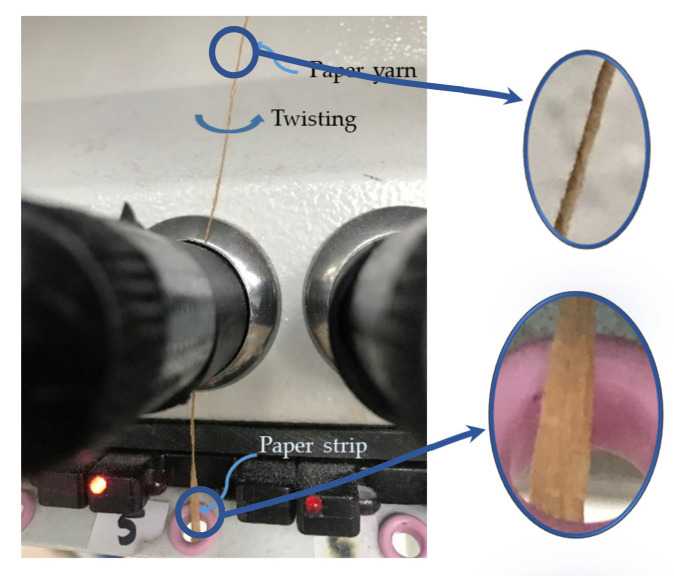
Production of paper yarn from a paper strip.

A Swedish paper yarn (AB Svenskt Konstsilke, Gothenburg, Sweden) with a linear density of 119 tex and a diameter of 0.35 mm was used for this study; the paper yarn production was done by cutting the paper sheets into strips with a width of 3 mm; then, these strips were twisted to form yarns (Figure 1), and the paper was moistened with water during the twisting process [19].

Six different finishing materials have been used. Three of them are silicon-based, since silicone is an efficient lubricating and soft handle finishing for textile [42,43,44,45]. One is glycerol, which is a sustainable textile fiber softener and is also used for paper coating [34,35,36,37,38,39], another one is wax, as it is a common lubricant used for yarn before knitting [33], and the last one is soap, since it has been used as a finishing material affecting the frictional properties of textiles [41]:

- The Finishing material 102 provided from GA Lindberg (Stockholm, Sweden) is an aqueous emulsion based on siloxanes and additives.
- Tubingal RGH (CHT n.d., Tübingen, Germany) is a micro emulsion of a polysiloxande organomodifier (CHT (Safety Data Sheet)). Tubingal RGH is a hydrophilic soft handle agent suitable for all fiber types.
- Ultratex^®^ UHS (Huntsman Corporation (HUN), TX, USA) is a micro emulsion of a functional polydimethyl siloxane. It is used as a hydrophilic finish and softener of different textile fibers.
- Glycerol from Merk KGaA (Darmstadt, Germany).
- Destofil LC Liquid is a weakly cationic wax dispersion used to improve the sliding properties of yarns of all types of fibers. The wax reduces the yarn-to-metal friction (Archroma n.d, Reinach, Switzerland).
- Soap: potassium carbonate, sodium sulfate, and water (Grumme, Orkla care, Stockholm, Sweden).

### 2.2. Finishing Application Method

**Figure 2:**
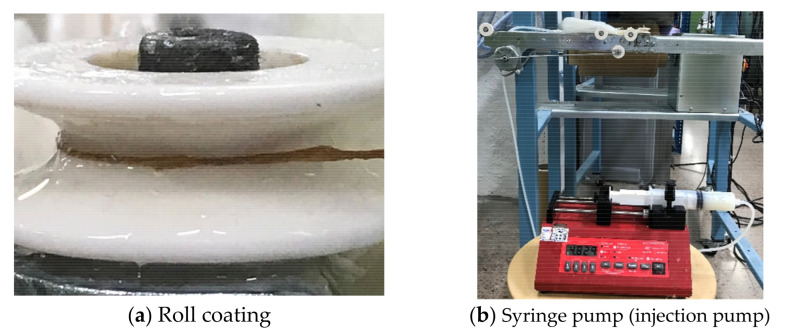
Finishing material application on yarn was made using (**a**) Roll coating where finishing material was applied to the yarn during movement; (**b**) Syringe pump used for injection of the finishing material at the targeted rate.

The application method was done using roll coating (Figure 2a). The application was done at 22 ± 2 °C with 42 ± 2% relative humidity. The injection rate was 1.2 mL/min by a syringe pump (injection pump AL-1000 WPI, World Precision Instruments, Sarasota, FL, USA) (Figure 2b), and the speed of the yarn during the finishing process was 25 m/min. For an even distribution of the finishing material, the winder speed had to be adapted for two finishing materials: 11 m/min for 102 and 14 m/min for Ultratex UHS.

The finishing materials were injected without any solvent, except for Ultratex UHS, which was diluted with 25 wt% water with a hardness of 3 °dH, pH of 8.1 at 24.1 °C, and alkalinity of 58 mg HCO_3_/L.

**Figure 3:**
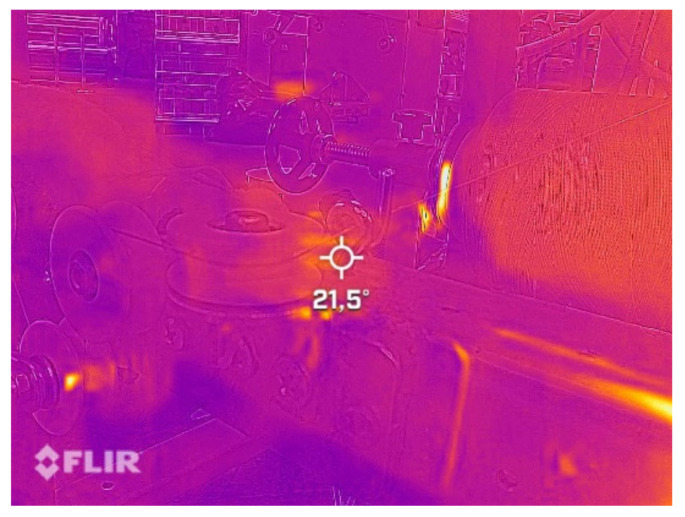
Measuring paper yarn temperature during the application of finishing materials using an infrared camera.

The yarn temperature was measured during the injection using a thermographic camera (infrared camera) FLIR ONE PRO (Teledyne FLIR, Tallinn, Estonia) to ensure that there were no changes in the yarn temperature during the application of finishing materials; see Figure 3.

The yarn was cured for 24 h after the application. After the drying, the finishing materials applied were studied by calculating the linear density of the yarn before and after treatment, where the yarn had been conditioned for 24 h at the standard atmosphere of 65 ± 2% relative humidity and 20 ± 2 °C before each measurement. Three measurements of 1 m yarn were made. The increase in weight percent denoted as *wt* after adding the coating material is given as follows.

(1) $$wt=100-\left(\frac{{\bar{m}}_{1}}{{\bar{m}}_{2}}\right)\times 100\%$$

In Equation (1), ${\bar{m}}_{1}$ and ${\bar{m}}_{2}$ are the average of linear density of the untreated and treated paper yarn (mg/m), respectively.

### 2.3. Coefficient of Metal/Yarn Friction Test

**Figure 4:**
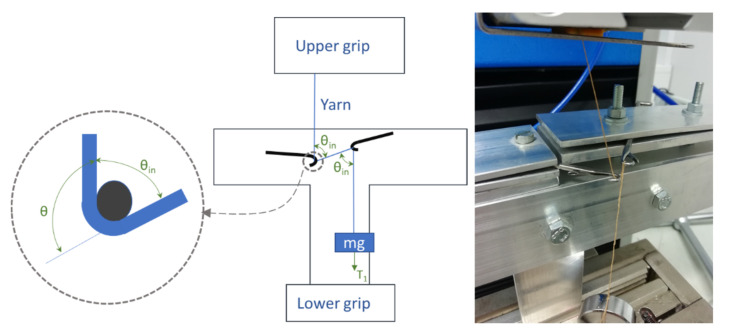
A schematic and a picture of the rig were used with a tensile tester to determine the coefficient of metal yarn friction.

To determine the coefficient of friction, a specially designed rig was used. The development of the rig is described in [46], and a schematic can be seen along with a picture in Figure 4. The method is a modification of the standard ASTM D3108-07 Standard Test Method for Coefficient of Friction, Yarn to Solid Material [47].

The objective of adapting the test method is to simulate the yarn/needle friction during the knitting process and use a tensile testing machine to determine the friction coefficient. Through using the same angle for yarn movement during the knitting process in this adaptation, the effect of the bending stiffness for paper yarn on the friction coefficient is integrated.

The test rig is mounted at the lower grip of the tensile tester. The sample is mounted at the upper grip with a pneumatic clamp and then lead through two latch needles. The latch needles used had a diameter of 1.4 mm and are of the type used in conventional flat knitting machines with the gauge of E4. The setup gave a cumulative wrap angle of 210°. A 5 g weight was attached to the loose end of the sample contributing to a 49.1 mN pre-load at testing. As the upper clamp moves, the yarn is dragged between the latch needles, and a friction force is built up between the yarn and needles. The clamp speed used was 100 mm/min. Three repetitions of each treatment combination were made after 24 h conditioning in a standard atmosphere of 65 ± 2% relative humidity and 20 ± 2 °C.

The coefficient of metal/yarn friction denoted as *µ* is given by Equation (2).

(2) $$\mu =\frac{\ln T_{2}-\ln T_{1}}{2\theta }$$

In Equation (2), *T*_1_ is the pre-tension force (mN), *T*_2_ is the applied force (mN) measured by the load cell, and *θ* is the cumulative wrap angle (rad). The cumulative wrap angle *θ* is given by Equation (3).

(3) $$\theta =\frac{2\pi }{360^{{}^{\circ}}}\left(180^{{}^{\circ}}-\theta_{in}\right)$$

In Equation (3), *θ_in_* is the angle of the yarn going around the needle, as shown in Figure 4.

### 2.4. Tensile Testing

For the tensile tests, a Tensolab 2512A/2512C electromechanical tensile tester (Mesdan, Puegnago del Garda, Italy) was used with pneumatic clamps and a 0.1 kN load cell. The testing was performed according to method A in the standard SS-EN ISO 2062:2009 Textiles—Yarns from packages—Determination of single-end breaking force and elongation at break using a constant rate of extension (CRE) tester [48]. The gauge length was 250 mm, the speed was 250 mm/min, and the pre-tension was 0.5 ± 0.1 cN/tex. Lack of material made it impossible to perform the correct number of samples according to the standard. Five repetitions were successfully tested from each treatment combination. Before testing, the samples were conditioned for 24 h in the standard atmosphere of 65 ± 2% relative humidity and 20 ± 2 °C.

### 2.5. Knitting Evaluation

Knitting evaluation was made by recording the number of stops and yarn breaks during the knitting. A Stoll CMS 822 HP knit and wear flat knitting machine (Lengede, Germany) was used with a multigauge of 5.2 at 35% of maximum speed for the knitting of samples in a single interlock. Three samples of each treatment were produced. A weft feeder from Iro Orion was connected to the knitting machine and provided an even yarn tension at knitting.

To make it possible to knit the untreated yarn, a water spray bottle was used to keep the water content of the yarn at about 28 wt%.

### 2.6. Microscopy

For evaluation of the knittability, a Dino-Lite Classic 200× USB microscope (Hsinchu, Taiwan) was used for the knitted samples. The pictures were analyzed to investigate if the yarn surface was changed due to the treatment.

## 3. Results

### 3.1. Yarn Coating

**Figure 5:**
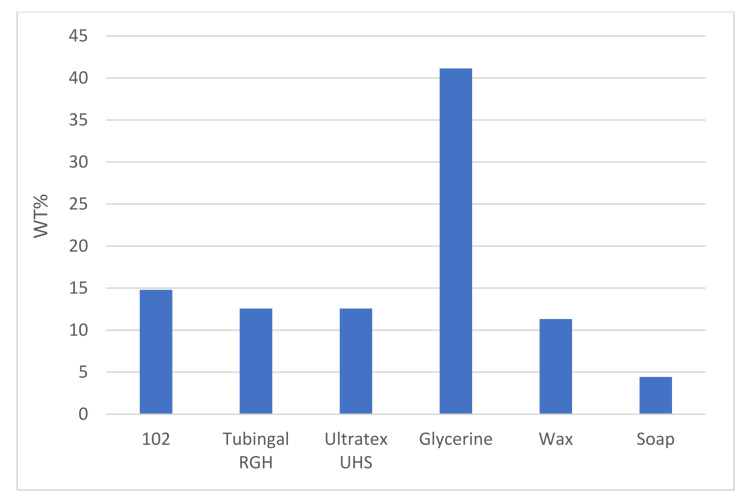
Increase in wt% of paper yarn due to the application of finishing materials.

The application of finishing materials resulted in increased yarn weight. Figure 5 show the resulting increase in wt% calculated from three replicas. The glycerol was highest with 41.14 wt%, and the soap resulted in the lowest weight increase, 4.43 wt%. For the other four finishing materials, the weight increase was between 11.32 and 14.80 wt%.

### 3.2. Coefficient of Metal Yarn Friction

**Figure 6:**
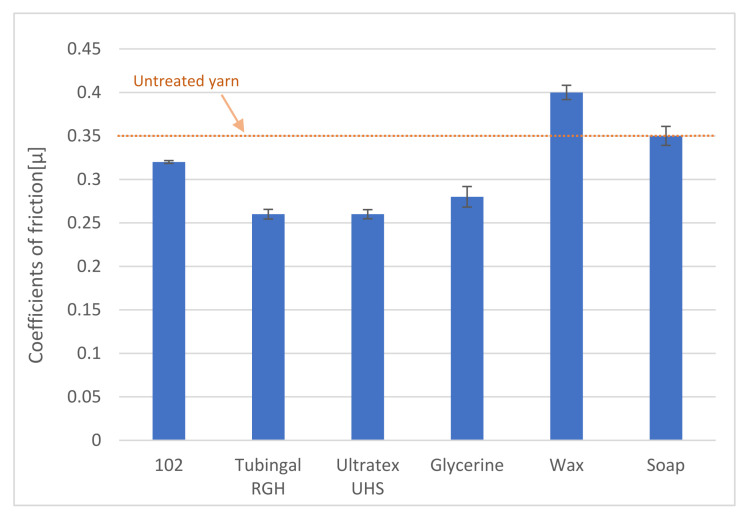
Coefficients of friction were determined for the paper yarn before and after applying finishing materials.

In Figure 6, the different coefficients of friction are shown. Soap treatment did not change the coefficient compared to untreated yarn, and wax resulted in an increased coefficient of friction; this result is similar to what was found by Koo [33]: that the wax does not reduce the metal yarn friction. The other four finishing material coatings resulted in a decreased coefficient of friction. Levine’s test was used to evaluate if one-way ANOVA could be used for statistical analysis, and it could. The one-way ANOVA with a 95% confidence interval showed statistically significant differences between the friction coefficients for the different coatings. Tukey’s post hoc test showed that five out of six finishing materials give significant differences to the untreated yarn. The soap did not show a statistically significant difference compared to the untreated yarn. Tubingal RGH, Ultratex UHS, and glycerol have the same effect on the coefficient of friction, which conforms to what was found in the literature [22,27,30,31,32].

### 3.3. Tensile Testing

**Figure 7:**
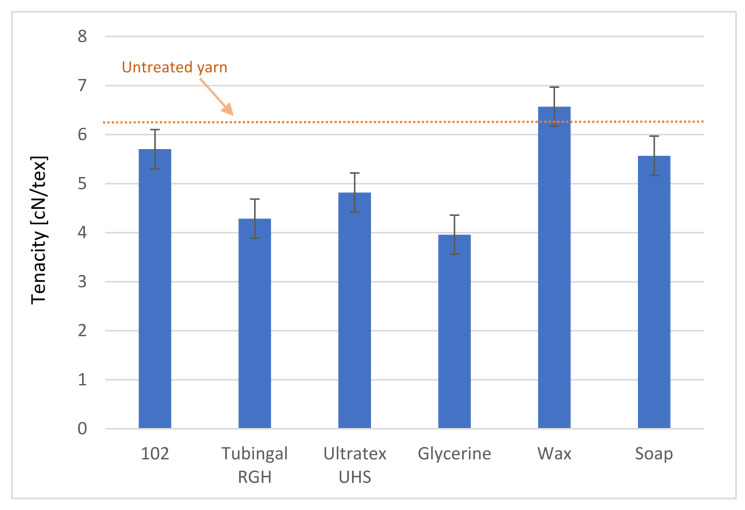
Effect of the finishing materials in the tenacity of paper yarn.

**Figure 8:**
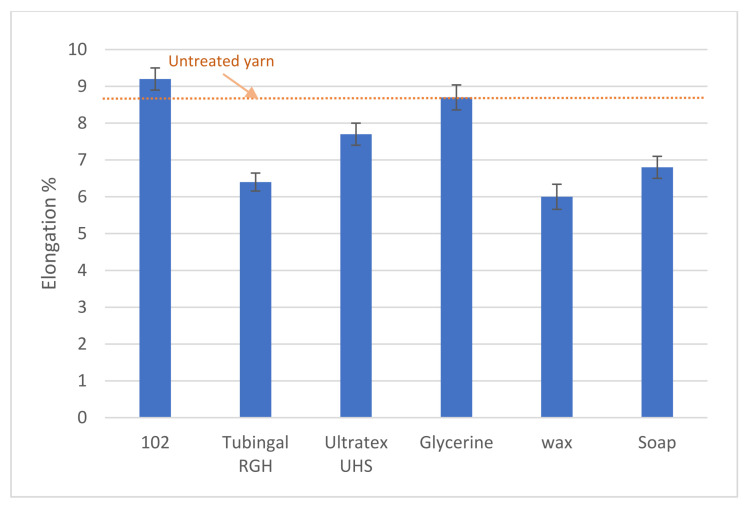
Effect of finishing materials in the elongation of paper yarn.

It is important that the finishing materials do not decrease the strength of the yarn, as it must be able to withstand the processing loads, and the end product properties need to be sufficient. The results from tensile testing are presented in Figure 7 and Figure 8. In Table 1, the changes are presented in percent. None of the tested finishing materials sustains tenacity, strength, and elongation. Tenacity is defined as the mass stress at break. Tenacity is common in the textile area, since yarn usually has a large variation in its cross-section area. Wax coating results in increased tenacity and strength, but the elongation is decreased by 31.03%. Wax was also the only coating leading to an increased coefficient of friction. Soap had a sustained coefficient of friction but decreased all tested mechanical properties. The glycerol, Tubingal RGH, and Ultratex UHS showed the most significant decrease in coefficient of friction, but they all show decreased values for the mechanical properties. This can be explained by the lubricant effect, which reduces the tenacity of the yarn [26,30].

The glycerol-treated samples had sustained elongation corresponding to its effect on the paper [36]. The finishing 102 increases the elongation by 5.74% but decreases the tenacity and strength of the yarn by 9.04% and 8.98%, respectively. The coefficient of friction for 102 treated samples was decreased from 0.35 to 0.32, which places it below soap and above the three samples: glycerol, Tubingal RGH, and Ultratex UHS.

### 3.4. Knitting Evaluation

During the knitting, the number of stops and yarn breaks were recorded. It was not possible to knit the untreated paper yarn in dry condition. Therefore, the water content of the untreated yarn was kept at about 28 wt% by spraying. The second in knitting difficulty after the untreated, dry yarn, was soap-coated yarn. Several stops occurred due to yarn breakage. The wax-treated yarn had a few stops, too. The knitting process was not inhibited by the yarn for any of the other samples: 102, glycerol, Tubingal RGH, Ultratex UHS, and untreated wet yarn.

Naked eye inspection of the knitted samples revealed a few knitting faults for some of the finishing materials. Several holes were found in the soap-finished samples due to lower elongation and tenacity associated with a high friction coefficient. A few holes were found in the wax samples, where the augmentation of tenacity can explain the lower number of holes even though the coefficient of friction for waxed paper yarn is the highest.

The samples with difficult knitting and resulting faults all belong to the top three in a high coefficient of friction. A paper yarn with a coefficient of friction below 0.35 was not difficult to knit. Wax and soap were difficult to knit, and besides their high friction coefficients, their elongations had decreased (31.03% and 21.83%, respectively).

However, the Tubingal RGH-treated sample was not difficult to knit, even though the mechanical properties had decreased a lot compared to the other samples: −31.62% tenacity, −31.63% strength, and −26.43% elongation, which can be explained by the high effect on the metal/yarn friction in the knitting process.

### 3.5. Microscopy

**Figure 9:**
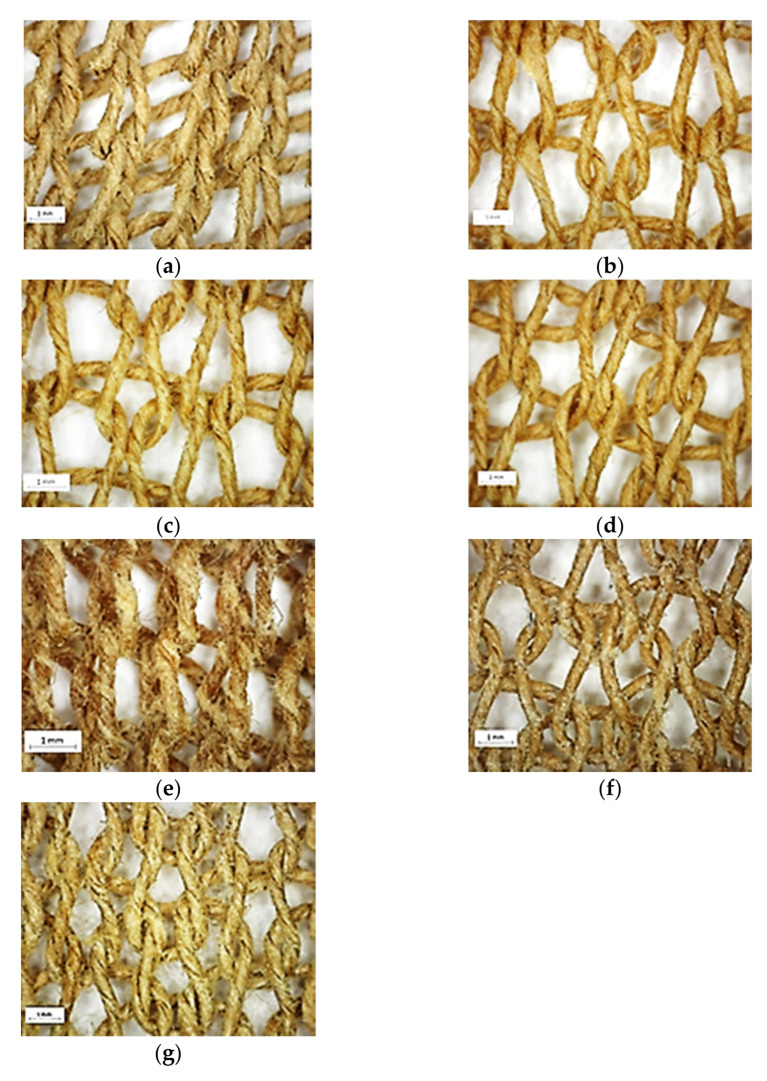
Microscopy pictures of the knitted samples. (**a**) Untreated yarn; (**b**) 102-finished yarn; (**c**) Tubingal RGH finished yarn; (**d**) Ultratex UHS finished yarn; (**e**) Glycerol finished yarn; (**f**) Wax finished yarn; (**g**) Soap finished yar.

The knitted samples were visually inspected by microscopy (Figure 9), showing that it is not possible to predict the knittability by the samples’ look. The three silicone-based finishing materials all lead to increased knittability compared to the dry untreated yarn; they also show a thin and sleek impression. Glycerol-coated yarn was also knittable but shows the roughest surface with a lot of protruding fibers. The untreated yarn was knittable after spraying with water, and the resulting knit shows a slightly swollen look of the yarn.

Neither wax nor soap-finished yarn was knittable. Wax shows a thin yarn similar to the silicone-treated samples, but the microscopy also revealed that the wax gathered in clusters at the yarn surface. This could be the reason for the knitting difficulties and its high coefficient of friction. Another reason could be that the adsorption of finishing material was lowest for wax and soap. The soap-coated yarn looks similar to the uncoated yarn, with slightly more protruding fibers. The coefficient of friction of soap-coated yarn was sustained compared to uncoated dry yarn; neither of them was easy to knit.

In summary, a sleek yarn is knittable if there are no clustered finishing materials at the surface. Glycerol-coated yarn is the exception, being knittable even though there are a lot of protruding fibers seen by microscopy.

## 4. Discussion

Renewability is essential for sustainable materials used in the textile industry, and paper is a renewable resource. However, many paper yarn properties require improvements to increase their processability. For knitting, friction is an important aspect, and it is usually modified in the textile industry through finishing (coating). The literature review showed that there are many factors influencing friction both individually and in combination.

Therefore, in this work, the paper yarn has been finished with different finishing materials commonly used in textiles processing. Their influence on the coefficient of friction and the knittability of Swedish paper yarn has been studied.

The literature on lubricant uptake states that dry application methods give less lubricant on sewing threads than wet application methods and that 2% lubrication could significantly decrease the friction, and it also states that too much lubrication is harmful to processing [28]. However, in our study, the uptake is much higher (up to 40%) than the literature values (4–6%). Although the knittability was poor for the two lowest uptake amounts, about 4% and 10%, the friction coefficient was not decreased accordingly to the amount of lubricants. This indicates that paper yarn can absorb a lot of finishing materials, leading to a lesser amount of materials ending at the surface. The most suitable amount is depending on the absorption of the finishing materials by the paper. The uptake could be managed by changing the paper sorption properties before yarn making.

The microscopy revealed that wax-treated yarn, with difficulties during knitting, had gathered finishing material clusters at the yarn surface. Hence, the optimum amount of lubricant might have been exceeded for wax in our work.

Property losses were expected, as seen in the literature. The property losses in our work are sometimes higher than those found in the literature, but we also see increases in some properties. These increases are not reported in the literature. It could be explained by the different yarn structures of paper yarn, where the mechanical properties are not governed by the individual fibers but by the paper structure.

The literature also states that silicone oil would be poor for the properties [26,30] where we get similar results for the tenacity and elongation. Some of the property decreases observed here were higher than those reported in the literature. This was not expected, as the paper yarn structure should make it stiffer and perhaps more resistant to the fiber–fiber lubrication seen for other materials in literature. Regardless of the resulting change in properties, it is unknown if the interaction mechanism between lubricant and yarn is different in paper yarn compared to regular yarns.

In our work, the yarn–metal friction was tested, but perhaps we see changes in fiber–fiber friction indirectly through the changes in properties. Studying the mechanisms of the interactions between finishing materials and textile materials is needed and was also suggested by Nadi 2018 [8]. Such an understanding would save time when choosing which finishing materials to use for which yarn.

As the friction coefficient is affected by many factors, each new material must be tested under its specific relevant conditions. In the literature, the coefficient of friction is said not to be less than 0.2 for lubricated yarns [22], and our results agree. None of the tested yarns showed a coefficient of frictions below 0.25.

From the results, it can also be added that the coefficient of friction should be below 0.35 for the knittability of this paper yarn. The friction is affected by the surface roughness, but according to the literature [23,24,25], the relationship can be material-dependent. This could explain the low coefficient of friction seen for glycerol-treated paper yarn, even though the surface roughness was deemed high using microscopy.

The one-way ANOVA shows statistically significant differences between the coefficient of friction means of the treatments. Tubingal RGH, Ultratex UHS, and glycerol all have the same effect on the coefficient of metal/paper yarn friction. Soap did not show any statistically significant effect on the coefficient of metal/paper yarn friction.

However, the coefficient of metal/paper yarn friction is not the only factor determining the processability, and other factors and properties such as tenacity would be interesting to investigate.

Further studies on the relationship between paper yarn surface roughness and coefficient of friction need more investigation to see if there is a minimum coefficient of friction for specific surface roughness. It will also be relevant to see if the coefficient of metal/paper yarn friction depends on the finishing treatment.

Our results of the present study show that a higher elongation and lower coefficient of friction increased the processability in knitting. To the list of flexibility-increasing substances, oil, waxes, and steaming previously mentioned in literature [11,13], we can now add silicone and glycerol.

Silicone-based finishing materials show a good result in enhancing the knittability, but it does not mean that it would be the best candidate for large-scale processing. The sustainability of silicone use should be considered, as several companies today look for replacements due to environmental reasons.

There is a significant advantage when the properties can be changed using non-toxic substances such as regular water. Water has previously shown good results in the spinning of Hanji paper yarn and now also in the knitting process of Swedish paper yarn [11]. The water effect in the paper properties should partly depend on the paper properties, such as wet strength.

High stiffness is a drawback, especially for knitting. Increased stretchability and elongation can improve paper yarn processing. Knitting is also greatly influenced by friction, as was investigated in this work. The ability of the yarn to form loops is also essential for knitting; therefore, it would be interesting for the future to investigate the influence of paper yarn flexibility on knittability. If paper yarn can be adapted to knitting, the application area would be broadened, and paper yarn could be a complementary fiber of a greater market share.

## 5. Conclusions

The knittability of the new Swedish paper yarn used in this study can be enhanced by silicone-based or glycerol finishing. The result of this study is that the coefficient of friction between paper yarn and metal can be reduced with silicone-based or glycerol finishing of the yarn. The idea for this comes from research of other cellulose yarns where it is used. The unique novelty of this here is the combination of these finishing materials, the paper yarn, and the enhanced knittability. This is an important step toward a new sustainable textile fiber to partly fill the future need of more fibers as the population grows. It has been shown that the paper yarn can be knitted, which opens up for additional product opportunities made from paper yarn.

The results show that the coefficient of friction between paper yarn and metal can be reduced with silicone-based or glycerol finishing. The knittability was enhanced for paper yarn finished with silicone or glycerol. It was also possible to knit with the untreated paper yarn with wetting. The finishing also affects the feel of the knit, where untreated yarn results in the roughest feeling.

The main drawback of the finishing materials used in this work is the negative effects on the mechanical properties of the yarn. Most of the tested finishing materials in this work reduce the strength of the paper yarn. The silicone-based finishing material named 102 increases the elongation by 5.7% but reduces the tenacity by 9.0% and the strength by the same value. Wax increases the strength and tenacity by 4.8%. However, the wax coating also resulted in knitting faults. Hence, it is not fulfilling the purpose of the treatment.

The drawbacks aside, it has been shown that the knittability of Swedish paper yarn can be improved by finishing materials. The findings demonstrate the potential of Swedish paper yarn in serving as a sustainable yarn in the textile and fashion industry.

For future work, it is suggested to study the mechanisms for the interaction between finishing materials and paper yarn textiles. The finishing treatments should be optimized in terms of the amount and application method, and also, the influence of treatment on the yarn flexibility needs more investigation.

## Figures and Tables

**Table 1:** The changes in tenacity, strength, and elongation for coated yarns.

Finishing Material	Δ-Tenacity [%]	Δ-Strength [%]	Δ-Elongation [%]
Reference	0	0	0
Wax	4.78	4.82	−31.03
Soap	−11.18	−11.12	−21.83
102	−9.04	−8.98	5.74
Glycerol	−36.85	−36.86	0
Tubingal RGH	−31.62	−31.63	−26.43
Ultratex UHS	−23.14	−23.19	−11.49

## Data Availability

Not applicable.

## References

[1] Aldalbahi A., El-Naggar M.E., El-Newehy M.H., Rahaman M., Hatshan M.R., Khattab T.A. (2021). Effects of technical textiles and synthetic nanofibers on environmental pollution. Polymers.

[2] Ferrándiz M., Fages E., Rojas-Lema S., Ivorra-Martinez J., Gomez-Caturla J., Torres-Giner S. (2021). Development and characterization of weft-knitted fabrics of naturally occurring polymer fibers for sustainable and functional textiles. Polymers.

[3] Karthik T., Gopalakrishnan D. (2014). Environmental Analysis of Textile Value Chain: An Overview.

[4] Laitala K., Klepp I.G., Henry B. (2018). Does use matter? Comparison of environmental impacts of clothing based on fiber type. Sustainability.

[5] Lakshmanan S.O., Raghavendran G. (2018). Regenerated Sustainable Fibres.

[6] Lee K.E. (2017). Environmental Sustainability in the Textile Industry.

[7] Luján-Ornelas C., Güereca L., Franco-García M.-L., Heldeweg M. (2020). A Life Cycle Thinking Approach to Analyse Sustainability in the Textile Industry: A Literature Review. Sustainability.

[8] Nadi A., Boukhriss A., Bentis A., Jabrane E., Gmouh S. (2018). Evolution in the surface modification of textiles: A review. Text. Prog..

[9] Patti A., Cicala G., Acierno D. (2020). Eco-sustainability of the Textile Production: Waste Recovery and Current Recycling in the Composites World. Polymers.

[10] TextileExchange (2020). Preferred Fiber & Materials in Market Report.

[11] Park T.Y., Lee S.G. (2013). A study on coarse Hanji yarn manufacturing and properties of the Hanji fabric. Fibers Polym..

[12] Sahlstrand M., Koplos J. (1984). Paper Clothing, East and West. Fiberarts Mag..

[13] Murate H., Terasaki F., Shigematsu M., Tanahashi M. (2008). Improvement in the Stretching Property of Paper Yarn by Shape Memorization Produced with High-pressure Steam Treatment. Sen’i Gakkaishi.

[14] Peterson J., Eckard A., Hjelm J., Morikawa H. (2019). Mechanical-Property-Based Comparison of Paper Yarn with Cotton, Viscose, and Polyester Yarns. J. Nat. Fibers.

[15] Rundberg S., Persson L. (2016). Pappersgarn i Mode. Bachelor’s Thesis.

[16] Amini A., Zohoori S., Mirjalili A., Karimi L., Davodiroknabadi A. (2014). Improvement in physical properties of paper fabric using multi-wall carbon nanotubes. J. Nanostruct. Chem..

[17] Park T., Kim M.-O. (2018). Manufacture and physical properties of the denim fabrics using Hanji paper yarn as weft yarn. Fash. Text..

[18] Vasell A., Ronkainen J. (2017). Mekanisk Mjukgöring av Pappersgarn: En Studie om Smärgling av Pappersgarn Samt Behandlingens Påverkan på de Taktila Egenskaperna. Bachelor’s Thesis.

[19] Chummun J., Rosunee S. (2012). Manufacture of Folded and Twisted Paper Yarn. Res. J. Text. Appar..

[20] Pan N. (1992). Development of a Constitutive Theory for Short Fiber Yarns: Mechanics of Staple Yarn without Slippage Effect. Text. Res. J..

[21] Pan N. (1993). Development of a Constitutive Theory for Short Fiber Yarns Part II: Mechanics of Staple Yarn with Slippage Effect. Text. Res. J..

[22] Ek M., Gellerstedt G., Henriksson G. (2009). Volume 1: Wood Chemistry and Wood Biotechnology. Pulp and Paper Chemistry and Technology.

[23] Ek M., Gellerstedt G., Henriksson G. (2009). Volume 2: Pulping Chemistry and Technology. Pulp and Paper Chemistry and Technology.

[24] Shahinur S., Ullah A.S. (2017). Quantifying the Uncertainty Associated with the Material Properties of a Natural Fiber. Procedia CIRP.

[25] Ashadujjaman M., Saifullah A., Shah D.U., Zhang M., Akonda M., Karim N., Sarker F. (2021). Enhancing the mechanical properties of natural jute yarn suitable for structural applications. Mater. Res. Express.

[26] Gurarda A., Yukseltan E., Kaplangiray B.M., Kanik M. (2013). The effects of various lubricants on the friction properties of sewing threads. Text. Res. J..

[27] Jawale S.N.P., Patil U.J. (2011). A Review on Yarn Friction—Importance, Theory, Factors & Measurement.

[28] Çukul D., Candan C., Turan S. (2010). Utilizing scanning electron. Microscopy stereoscopy to explain the wear behavior of latch needles. Text. Res. J..

[29] Eryuruk S.H., Kalaoglu F. (2010). The effects of different amounts of lubricant application on the sewing thread performance properties. Text. Res. J..

[30] Senthil N., Dhurai B. (2021). Knittability Enhancement Study of 100% Linen yarn using Softeners. J. Nat. Fibers.

[31] Mengüç G.S., Özgüney A.T., Dalbaşi E.S., Özdil N. (2019). A comparative study on handle properties of bamboo and cotton fabrics. Ind. Text..

[32] Shablygin M.V., Petrova M.V., Sklyarova G.B., Kashirina M.A. (2014). Nature of Interaction of Lubricating Composites with Para-Aramid Yarns. Fibre Chem..

[33] Koo Y.S. (2008). Waxing Effect on Lint Contamination in the Knitting Process. Text. Res. J..

[34] Ferrero F., Periolatto M. (2012). Glycerol in comparison with ethanol in alcohol-assisted dyeing. J. Clean. Prod..

[35] The Soap and Detergent Association (1990). Glycerine: An Overview.

[36] Gu Y., Jérôme F. (2010). Glycerol as a sustainable solvent for green chemistry. Green Chem. Int. J. Green Chem. Resour. GC.

[37] Alvarez M.G., Segarra A.M., Contreras S., Sueiras J.E., Medina F., Figueras F. (2010). Enhanced use of renewable resources: Transesterification of glycerol catalyzed by hydrotalcite-like compounds. Chem. Eng. J..

[38] Behr A., Eilting J., Irawadi K., Leschinski J., Lindner F. (2007). Improved utilisation of renewable resources: New important derivatives of glycerol. Green Chem..

[39] Wolfson A., Dlugy C., Shotland Y. (2006). Glycerol as a green solvent for high product yields and selectivities. Environ. Chem. Lett..

[40] Aloui H., Khwaldia K., Ben Slama M., Hamdi M. (2011). Effect of glycerol and coating weight on functional properties of biopolymer-coated paper. Carbohydr. Polym..

[41] Mondal S., Reddy V., Sarkar A., Aravindakshan P., Ghatak A. (2016). Effect of surface modification on frictional properties of polyester fabric. Tribol. Int..

[42] Wei Y., Zheng C., Chen P., Yu Q.J., Mao T., Lin J., Liu L. (2018). Synthesis of multiblock linear polyether functional amino silicone softener and its modification of surface properties on cotton fabrics. Polym. Bull..

[43] Zia K.M., Tabassum S., Barkaat-Ul-Hasin S., Zuber M., Jamil T., Jamal M.A. (2011). Preparation of rich handles soft cellulosic fabric using amino silicone based softener. Part-I: Surface smoothness and softness properties. Int. J. Biol. Macromol..

[44] Xu Y., Yin H., Zheng H., Yuan S., Chen Z. (2010). Application performance and surface morphologies of amino polysiloxanes with different amino values and amino types. J. Appl. Polym. Sci..

[45] Abolhasani M.M., Jalaei A., Tavana R., Kashani F.Z. (2019). Processing and performance properties of amino silicone-based softener on various textile substrates. Polym. Bull..

[46] Peterson J., Vegborn E. (2009). Development of a pre-knitting friction test method and study of friction and bending of yarns with high stiffness. Magisteruppsats.

[47] ASTM (2020). D3108/D3108M Standard Test Method for Coefficient of Friction, Yarn to Solid Material.

[48] (2009). ISO 2062:2009 Textiles—Yarns from Packages—Determination of Single-End Breaking Force and Elongation at Break Using a Constant Rate of Extension (CRE) Tester.

